# Role of Age-Related Mitochondrial Dysfunction in Sarcopenia

**DOI:** 10.3390/ijms21155236

**Published:** 2020-07-23

**Authors:** Evelyn Ferri, Emanuele Marzetti, Riccardo Calvani, Anna Picca, Matteo Cesari, Beatrice Arosio

**Affiliations:** 1 Geriatric Unit, Fondazione IRCCS Ca’ Granda Ospedale Maggiore Policlinico, 20122 Milan, Italy; matteo.cesari@unimi.it (M.C.); beatrice.arosio@unimi.it (B.A.); 2 Fondazione Policlinico Universitario “Agostino Gemelli” IRCCS, 00168 Rome, Italy; emarzetti@live.com (E.M.); riccardo.calvani@gmail.com (R.C.); anna.picca1@gmail.com (A.P.); 3 Geriatric Unit, Università Cattolica del Sacro Cuore, 00168 Rome, Italy; 4 Department of Clinical Sciences and Community Health, University of Milan, 20122 Milan, Italy

**Keywords:** skeletal muscle, muscle aging, sarcopenia, mitochondria, mitochondrial dysfunction

## Abstract

Skeletal muscle aging is associated with a significant loss of skeletal muscle strength and power (i.e., dynapenia), muscle mass and quality of life, a phenomenon known as sarcopenia. This condition affects nearly one-third of the older population and is one of the main factors leading to negative health outcomes in geriatric patients. Notwithstanding the exact mechanisms responsible for sarcopenia are not fully understood, mitochondria have emerged as one of the central regulators of sarcopenia. In fact, there is a wide consensus on the assumption that the loss of mitochondrial integrity in myocytes is the main factor leading to muscle degeneration. Mitochondria are also key players in senescence. It has been largely proven that the modulation of mitochondrial functions can induce the death of senescent cells and that removal of senescent cells improves musculoskeletal health, quality, and function. In this review, the crosstalk among mitochondria, cellular senescence, and sarcopenia will be discussed with the aim to elucidate the role that the musculoskeletal cellular senescence may play in the onset of sarcopenia through the mediation of mitochondria.

## 1. Introduction

Declines in skeletal muscle mass and function are among the most notable corollary of aging. Muscle mass reaches its peak between 30 and 40 years of age and starts declining thereafter [1]. Such a decline is considered a normal phenomenon during aging, but it can rapidly progress in physically inactive persons as well as in the setting of acute or chronic conditions. In particular, in people with an inactive lifestyle, the loss of muscle mass can reach 1% to 2% per year from age 50 to 60, and 3% to 5% per year at older ages [2]. As a result, an inactive person can lose from 30% to 50% of muscle mass between the ages of 40 and 80 years [2].

The term sarcopenia was coined by Rosenberg [3] from the Greek words “sarx” (flesh) and “penia” (poverty) to describe the loss of muscle mass with aging. This condition affects nearly one-third of the older population [2], and is one of the main factors leading to negative health outcomes in older adults [4]. Over the years, sarcopenia has been better defined through the inclusion of reduced muscle strength and/or function (i.e., dynapenia) [5,6,7] in the conceptual framework [8,9,10,11,12]. Indeed, sarcopenia has recently been formally recognized as a disease by means of a novel ICD-10-MC code [13].

Several cellular and molecular mechanisms are involved in the pathogenesis of age-related muscle wasting. During aging, a decrease in the number and size of muscle fibers occurs, a process defined by Lynch et al. [14] “remodeling of the motor unit in relation to age”. Fiber atrophy and demise, in turn, result from reduced protein synthesis [15] and impaired muscle regeneration [16]. Other factors that contribute to muscle loss in advanced age include neuromuscular junction dysfunction, reduced satellite cell number/function, decreased number of motor units [17], intramuscular adipose tissue infiltration [18], inflammation [19], insulin resistance [20], mitochondrial dysfunction [21], and oxidative stress [22].

Although the exact mechanisms responsible for the development and progression of sarcopenia are not fully understood, mitochondrial dysfunction has emerged as a central pathogenetic factor [23]. Indeed, mitochondria serve a number of vital functions within the cell, including energy production, regulation of intracellular calcium homeostasis, modulation of cell proliferation, and integration of apoptotic signaling. Hence, the preservation of well-functioning mitochondria is pivotal for maintaining cellular homeostasis [24]. When mitochondrial quality control fails, mitochondria lose their integrity and may cause muscle degeneration [25]. Notably, the accumulation of damaged mitochondria has shown to trigger motor neuron and muscle fiber death, highlighting their relevance in the development of sarcopenia [26].

Here, we summarize available evidence supporting mitochondrial dysfunction as a mechanism contributing to musculoskeletal aging and sarcopenia. We also illustrate the involvement of mitochondria in cellular senescence with the aim to highlight the relationship between musculoskeletal cellular senescence induced by mitochondrial dysfunction and the onset of sarcopenia.

## 2. Mitochondria and Aging

Several evolutionarily conserved biological pathways have been indicated as the main drivers of aging [27]. Such pathways, collectively known as hallmarks of aging, include mitochondrial dysfunction, genomic instability, telomere attrition, epigenetic alterations, loss of proteostasis, deregulated nutrient-sensing, cellular senescence, stem cell exhaustion, and altered intercellular communication [27]. Previous studies showed that mitochondrial dysfunction arising from the abnormal accumulation of mitochondrial DNA (mtDNA) induced the early appearance of several age-related phenotypes, including sarcopenia, in mice [28,29,30]. However, whether laboratory rodents genetically engineered to accumulate lower amounts of mtDNA mutations during aging are protected against sarcopenia remains to be proven.

Researchers have proposed several theories to explain the functional decline associated with age-related mitochondrial dysfunction (Table 1). In particular, the notion that a shift in redox status towards oxidation leading to progressive cellular decline has gained momentum [31]. In this regard, when the defense system is no longer able to cope with the enhanced rate of oxidant production, cellular, and subcellular environments become more susceptible to damage [31]. For this reason, older persons activate a compensatory mechanism of upregulation of antioxidant enzymes to counteract the increasing generation of reactive oxygen species (ROS) [32]. However, despite the overactivation of antioxidant pathways, a positive correlation between age and oxidative damage is well-established with advancing age [33]. The persistence of sustained oxidative stress in spite of upregulated antioxidant activity suggests that antioxidant defense mechanisms may be overwhelmed in advanced age [33].

MtDNA copy number also varies during aging and might be considered a biomarker that mirrors alterations within the aged human body [34,35,36]. A new quantitative, highly sensitive droplet digital PCR method has allowed observing a mild gradual age-related reduction of the mtDNA copies in stimulated peripheral blood mononuclear cells in a sample ranging in age between 23 and 113 years [37]. Interestingly, centenarians with lower levels of frailty showed a significantly higher number of mtDNA molecules and fewer mtDNA deletions compared with those with more severe frailty [37].

## 3. Mitochondria and the Skeletal Muscle

Skeletal muscle is highly represented in the human body and is responsible for intentional movements and postural maintenance [42,43]. It has also a crucial role in some less obvious processes, such as thermal regulation, nutritional balance, glucose uptake, and endocrine activity [44,45].

The skeletal muscle of humans and other mammals consists of different cell types, i.e., multinucleated myofibers (or myotubes) and satellite cells, all wrapped in the sarcolemma [46,47]. A single myofiber is a postmitotic highly differentiated cell, which contains numerous peripheral nuclei, myofilaments, the sarcoplasmic reticulum, and finally the mitochondria, which represent the actual machinery providing energy to the movement [48].

### 3.1. Mitochondria Localization in Skeletal Myofibers

Ultrastructural studies have identified in human and rodent muscles different subsets of mitochondria localized within the skeletal myotubes: subsarcolemmal, perinuclear, and intermyofibrillar mitochondria [49,50,51]. Every subtype of mitochondria has peculiar biochemical and proteomic specializations. In particular, subsarcolemmal mitochondria have a role in gene expression and resistance to ROS, whereas intermyofibrillar mitochondria are concerned with processes such as oxidative phosphorylation and the modulation of Ca^2+^ flux [52]. Para-vascular mitochondria are specific to vessels, but have also recently been found in skeletal myocytes [53]. The peculiarities of human skeletal muscle mitochondria are their dynamic behavior within the myofiber [54], and their connectivity and/or ramification in the muscle, that is determined by mtDNA and could be subject to change in response to aerobic oxidative metabolism [55,56].

Two different types of skeletal muscle fibers are known according to the different isoforms of structural proteins: the myosin heavy chain and the tropomyosin [57]. The most common are type I or slow-twitch myofibers, and type II, or fast-twitch myofibers. This last type is further divided into type II A and type II X [58,59]. Red muscles mainly consist of slow types II A and I fibers and rely mostly on aerobic oxidative metabolism, while white muscles are made up of fast type II B fibers and adopt glycolysis [60,61]. Interestingly, in anaerobic glycolytic fibers, mitochondria are associated with the sarcomere I-band, whereas in oxidative fibers, mitochondria are mainly accumulated in I-band and A-band [62]. Moreover, in fast-oxidative or fast intermediate myofibers which can be found in red muscles, all triads are associated with mitochondria causing Ca^2+^ release from sarcoplasmic reticulum and ATP production [63,64].

### 3.2. Mitochondria Dynamics in the Skeletal Muscle

Changing metabolic demands induce modifications in the shape and dynamics of mitochondria residing in skeletal muscle [65]. A constant balance is maintained between the amount of short and elongated fused mitochondria. This balance results in fusion and fission processes as well as on the activity of shaping proteins [66,67]. A major process regulating metabolic plasticity is mitophagy [68]. The latter involved an organelle-specific form of macro-autophagy that drives dysfunctional mitochondria towards degradation. The quality control system of mitophagy guarantees the maintenance of the cellular structure and mitochondrial integrity. Mitochondrial fission precedes mitophagy: elevated ROS levels and loss of mitochondrial membrane potential are two key events triggering mitophagy [69]. Mitophagy and fusion/fission events are dysregulated when muscle atrophy develops [70], during which muscle protein degradation is accentuated [71,72]. In such circumstances, mitochondria become shorter and fragmented, and mitophagy flux is upregulated [73,74].

## 4. Mitochondria and Sarcopenia

**Figure 1:**
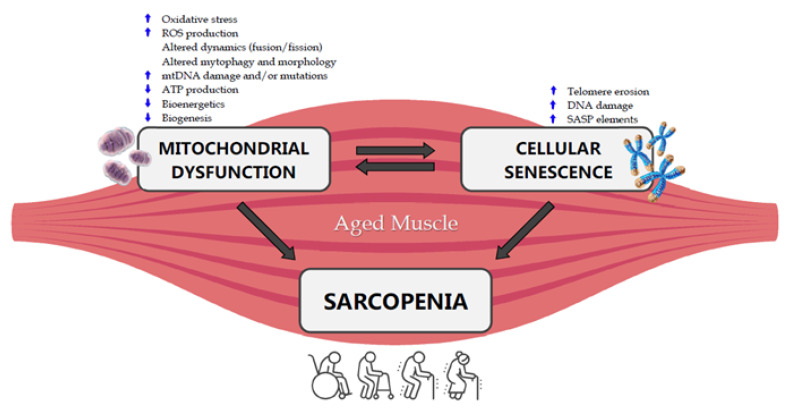
Crosstalk between mitochondrial dysfunction, cellular senescence and sarcopenia in the aged muscle. ROS: Reactive Oxygen Species, mtDNA: mitochondrial DNA, ATP: Adenosine Triphosphate, SASP: Senescence-Associated Secretory Phenotype.

Mitochondria have been indicated as the main actors in the development of sarcopenia (Figure 1) [25,75].

The absence of mtDNA histones and the lack of an efficient proofreading system are thought to be responsible for a progressive increase of somatic mtDNA mutations over the life course [27]. The load of mtDNA mutations and deletions is substantial in muscle fibers mostly affected by sarcopenia [76]. The accrual of mtDNA damage results in the synthesis of dysfunctional components of the electron transport chain, which in turn leads to defective ATP production and further ROS generation [31].

Another aspect to take into account is the central position of mitochondria in the regulation of apoptosis. Indeed, these organelles are involved in the integration of both intrinsic and extrinsic apoptotic pathways [77]. Notably, in mtDNA-mutator mice, the accumulation of mtDNA mutations is associated with and, perhaps, responsible for the upregulation of apoptotic signaling in several tissues, including the muscle [28]. This finding is in keeping with the idea that the enhancement of myonuclear apoptosis in the aging muscle may be due to mitochondrial dysfunction and oxidative stress [78].

Reduced activity of the major regulator of mitochondrial biogenesis, i.e., the peroxisome proliferator-activated receptor gamma coactivator-1α (PGC1-α) [79], may partially explain the altered mitophagy [80] and the decreased inactivity of cytochrome C oxidase observed in sarcopenia [81]. Mitochondrial biogenesis is a multistage process involving changes in the expression of more than 1000 genes and the activation of several transcriptional coactivators, to generate newly synthesized organelles. Several factors (e.g., inactive lifestyle, fasting, oxidative stress, inflammation) can negatively affect mitochondrial biogenesis [82].

Another major consequence of age-related mitochondrial dysfunction is the progressive decline in mitochondrial bioenergetics that is manifested by a reduction in maximum oxygen uptake [83]. In the aged skeletal muscle, the decrease in the number and function of mitochondria correlates with bioenergetics insufficiency [69]. In this regard, PGC-1α has been described not only as a master regulator of mitochondrial biogenesis but also as a mediator of the transcriptional outputs. Further, adenosine monophosphate-activated protein kinase (AMPK) and silent mating type information regulation 2 homolog sirtuin 1 (SIRT1), two of the best known metabolic sensors, can directly affect PGC-1α activity through phosphorylation and deacetylation, respectively. Insights from in vivo transgenic models clearly suggest that AMPK, SIRT1, and PGC-1α might act as an orchestrated network to control cellular energy expenditure and improve metabolic fitness [84].

PGC-1α may also prevent muscle atrophy through the regulation of autophagy [85]. With aging, PGC-1α levels dramatically fall in the skeletal muscle [86], while the maintenance of PGC-1α expression preserves muscle mass during sarcopenia, cachexia, denervation, and fasting [85,87]. This phenomenon seems to be mediated by the promotion of mitochondrial turnover and quality control [85,87].

### Role of Physical Training in Sarcopenia

The concerted activation of PGC-1α and SIRT1, co-localized in mitochondria, seems to be downstream of AMPK signaling in response to muscle contraction [88]. The interaction between PGC-1α and SIRT1 also suggests a role for SIRT1 in exercise-induced mitochondrial biogenesis [89]. Indeed, moderate long-term exercise stimulates metabolic adaptations in aged skeletal muscle through the activation of PGC-1α, AMPK, and SIRT1 pathways [90]. In fact, exercise training induces AMPK activation through the elevation of AMP/ATP ratio [91], which in turn induces mitochondrial biogenesis via PGC-1α activation [92].

These observations confirm the hypothesis that endurance exercise training may affect a specific set of functions (e.g., oxidative metabolism and mitochondrial biogenesis) and overall muscle metabolism [93]. Studies have shown that the skeletal muscle of older individuals undergoing an intense session of physical exercise produces a large amount of ROS [94]. At the same time, regular exercise tends to maintain low levels of oxidative damage and prevents sarcopenia [94]. It is likely that the excess ROS generated by boosts of physical exercise promotes the upregulation of antioxidant capacities, as a sort of "oxidative stress vaccination" [95]. Indeed, the stress imposed by exercise training is recognized as the most effective stimulus for mitochondrial biogenesis as part of redox-sensitive adaptation [96,97,98], resulting in enhanced mitochondrial function across the life course [99,100].

To date, exercise training is one of the best examples of mitohormesis, which is the phenomenon that occurs when an acute exposure to stress stimulates adaptive mitochondrial responses improving mitochondrial function and resistance to stress [101]. This implies that exercise training exerts a mitohormetic effect, positively influencing the maintenance and improvement of mitochondrial function, the antioxidant capacity, and the proteostasis. In turn, these adaptations contribute to the prevention of the age-related decline in skeletal muscle function, improving strength and muscle mass [33,101].

## 5. Mitochondria, Cellular Senescence and Sarcopenia

Cellular senescence is one of the most discussed mechanisms of aging. It can potentially be used to explain the cellular and molecular background at the basis of the muscle loss occurring with aging. At first, cellular senescence appeared to mainly be a consequence of the telomere shortening [102], but this assumption has gradually been evolved by numerous studies showing that the senescent phenotype derived also from other stresses such as oxidative stress, genomic damage, and activation of inflammatory features (Figure 1) [103].

Interestingly, mitochondria altered in function and morphology, and responsible for high levels of ROS, were found in senescent muscular cells [104]. In particular, impaired mitochondrial fission/fusion processes seem to affect the cell’s ability to degrade dysfunctional mitochondria, causing ROS-induced DNA damage and senescence [105]. Many studies associate the presence of a great amount of mitochondrial ROS with accumulated single-strand breaks in telomere regions, thus accelerating telomere erosion and cellular senescence [106,107]. Counteracting mitochondrial ROS generation, the rate of telomere shortening decelerates, the lifespan of muscular cells is extended and the muscle homeostasis is restored [104,108,109], delaying the onset of sarcopenia.

Additionally, several studies have shown that senescent cells accumulated in numerous aged tissues [110,111,112] may contribute to the worsening of the chronic inflammation status underlying the aging process (i.e., inflammaging) [113]. In turn, the inflammaging may contribute to muscle decline by impairing stem cell function and accelerating cellular senescence [113]. Evidence has shown that mitochondrial interventions at multiple regulation steps of the electron transport chain induce a senescent-like phenotype lacking the expression of pro-inflammatory senescence-associated secretory phenotype (SASP) elements, e.g., IL-6 and IL-8 [114,115]. Many of these pro-inflammatory cytokines have been found to alter the gene expression program of satellite cells, deeply affecting muscle regeneration [116] and contributing to the age-dependent decline in muscle function [117]. Moreover, a moderate musculoskeletal inflammatory status is able to induce muscle catabolism, a phenomenon particularly enhanced in cachexia and sarcopenia [118].

## 6. Conclusions 

Sarcopenia is a complex geriatric condition that is associated with a variety of negative health-related outcomes. Noticeably, the age-related muscle wasting is potentially preventable and treatable, which has instigated a growing interest around its pathophysiology. Several processes, both systemic and muscle-specific, have been shown to play a role in the pathogenesis of sarcopenia. Yet, a full comprehension of the etiology of sarcopenia is far from being reached. In this complex scenario, mitochondrial dysfunction in skeletal myocytes is recognized as a major driver of sarcopenia. Further research is necessary to understand whether mitochondrial dysfunction in muscle arises from primary organelle defects or defective quality control. Moreover, the contribution of systemic processes (e.g., inflammation) to muscle mitochondrial dysfunction remains to be fully elucidated. Answers to these open research questions will enable the development of targeted, person-tailored interventions against one of the most burdensome conditions of old age.

## Figures and Tables

**Table 1:** The Main Mitochondrial Theories of Aging.

Theory	Main Findings	Reference
The Free Radical Theory of Aging	The aging process is caused by cumulative oxidative damage to cells by free radicals	Harman [31]
The Superoxide Theory	Superoxide dismutase is an antioxidant defense against superoxide, the origin of most reactive oxygen species (ROS)	McCord and Fridovich [38]
The Oxidative Stress Theory	Oxidative stress is defined as an excessive accumulation of pro-oxidative features and ROS	Sies and Cadenas [39]
The Mitochondrial Free Radical Theory of Aging	Mitochondria is the main source of free radicals and the key target for oxidative damage	Miquel et al. [40]
The Free Radical Theory of Frailty	Oxidative damage does not correlate with chronological age but rather with their frailty state	Vina et al. [41]
